# Cutaneous Injury-Related Structural Changes and Their Progression following Topical Nitrogen Mustard Exposure in Hairless and Haired Mice

**DOI:** 10.1371/journal.pone.0085402

**Published:** 2014-01-08

**Authors:** Neera Tewari-Singh, Anil K. Jain, David J. Orlicky, Carl W. White, Rajesh Agarwal

**Affiliations:** 1 Department of Pharmaceutical Sciences, Skaggs School of Pharmacy and Pharmaceutical Sciences, University of Colorado Denver, Aurora, Colorado, United States of America; 2 Department of Pathology, School of Medicine, University of Colorado Denver, Aurora, Colorado, United States of America; 3 Department of Pediatrics, School of Medicine, University of Colorado Denver, Aurora, Colorado, United States of America; University of California Irvine, United States of America

## Abstract

To identify effective therapies against sulfur mustard (SM)-induced skin injuries, various animals have been used to assess the cutaneous pathology and related histopathological changes of SM injuries. However, these efforts to establish relevant skin injury endpoints for efficacy studies have been limited mainly due to the restricted assess of SM. Therefore, we employed the SM analog nitrogen mustard (NM), a primary vesicating and bifunctional alkylating agent, to establish relevant endpoints for efficient efficacy studies. Our published studies show that NM (3.2 mg) exposure for 12–120 h in both the hairless SKH-1 and haired C57BL/6 mice caused clinical sequelae of toxicity similar to SM exposure in humans. The NM-induced cutaneous pathology-related structural changes were further analyzed in this study and quantified morphometrically (as percent length or area of epidermis or dermis) of skin sections in mice showing these lesions. H&E stained skin sections of both hairless and haired mice showed that NM (12–120 h) exposure caused epidermal histopathological effects such as increased epidermal thickness, epidermal-dermal separation, necrotic/dead epidermis, epidermal denuding, scab formation, parakeratosis (24–120 h), hyperkeratosis (12–120 h), and acanthosis with hyperplasia (72–120 h). Similar NM exposure in both mice caused dermal changes including necrosis, edema, increase in inflammatory cells, and red blood cell extravasation. These NM-induced cutaneous histopathological features are comparable to the reported lesions from SM exposure in humans and animal models. This study advocates the usefulness of these histopathological parameters observed due to NM exposure in screening and optimization of rescue therapies against NM and SM skin injuries.

## Introduction

Upon cutaneous exposure, only 20% of sulfur mustard (SM) is absorbed, leading to acute and chronic skin lesions [1]. This warfare agent is a weapon of mass destruction, and its toxic skin effects have been observed in the Iranian soldiers and civilians exposed to SM during the Iran-Iraq conflict [2]–[4]. Depending upon the dose and time of SM exposure, within hours, edema develops and blister formation occurs. This is followed by ulceration, necrosis, desquamation, and pigmentation changes, dry skin after weeks and even years of exposure [5], [6]. The histopathology of these human skin lesions show signs of toxicity including pyknotic nuclei and death of some of the basal keratinocytes, and epidermal-dermal separation [7]. Other epidermal changes included parakeratosis, hyperkeratosis, hyperplasia, necrosis, apoptosis, acanthosis and multinucleated giant cells, and pigmentation changes [3], [4], [8], [9]. In addition to epidermal necrosis, dermal necrosis is also observed with infiltration of inflammatory cells, perivascular edema, vascular dilation, and extravasated erythrocytes [4], [8]. Animal models showing these toxic lesions by SM are crucial to understand the mechanisms underlying these effects, and to identify and develop effective treatments for SM-induced skin injuries.

Several animal models display many cutaneous lesions and structural changes with SM that are seen in humans [5], [8], [10]–[13]. However, there is still a limitation of access to mechanistic examination and routine laboratory efficacy studies in SM models due to its confinement to highly regulated containment facilities. Accordingly, our studies were directed to develop a practical experimental model and establish quantitative endpoints with the easily accessible SM analog of nitrogen mustard (NM). Both SM and NM are vesicating and powerful alkylating agents, and show parallel histopathological and immunohistochemical toxic effects following cutaneous exposure [6], [14]. However, comprehensive studies on the histopathological effects of NM skin injury in animal models that parallel SM exposure and can be subjected to useful efficacy studies are few. Our reported clinical observations with topical NM (3.2 mg) exposure in both SKH-1 hairless and C57BL/6 mice displayed clinical lesions and their progression, which were comparable to those reported in humans and other animal models with SM [15]. Our earlier report also presented clinically-relevant cutaneous injury endpoints that could be useful for efficacy studies to identify therapies against vesicating agents.

In the current study, we sought to further examine the histopathological effects and structural changes responsible for the clinical sequelae of NM-induced toxicity observed in our earlier reported study. Similar to this reported study, both hairless (SKH-1) and haired (C56BL/6) mice were used in this study to analyze the NM-induced histopathological changes. The current assessment will help define any differences in the injury response due to uptake or absorption of NM in the skin of both strains. This study demonstrates that NM cutaneous exposure resulted in epidermal thickness, epidermal-dermal separation, necrolytic/dead epidermis, epidermal denudation and ulceration, scab formation, parakeratosis, hyperkeratosis, and acanthosis associated with hyperplasia in both haired and hairless mice. NM exposure in these mice also caused dermal necrosis and edema, increase in inflammatory cells, and red blood cell extravasation. Overall, NM-induced structural changes in hairless and haired mice evaluated in this study parallel the reported lesions from SM exposure in humans and animal models.

## Materials and Methods

### Animals and NM Exposure

Both SKH-1 hairless and C57BL/6 (haired) mice (5–6 weeks of age) were purchased from Charles River Labs (Wilmington, MA), and housed under standard conditions and acclimatized before their use in our experimental studies. The Institutional Animal Care and Use Committee (IACUC) of the University of Colorado Denver, CO have specifically approved the studies with mice reported in this article. Mice (n = 5) were either exposed topically to 200 µL acetone alone or to 3.2 mg NM (mechorethamine hydrochloride; Sigma-Aldrich Chemical Co., St. Louis MO) in 200 µL acetone for 12 h, 24 h, 72 h and 120 h. Respective untreated control group was included in the study. C57BL/6 mice were shaved 3 days prior to NM exposure. Following the above mentioned exposures, mice were euthanized, the dorsal skin was collected and either snap frozen in liquid nitrogen or fixed in 10% phosphate buffered formalin as detailed earlier [15]–[17].

### Histopathological Evaluation

The formalin-fixed dorsal skin sample was dehydrated in ascending concentration of ethanol, cleared in xylene, and embedded in PolyFin (Triangle Biomedical Sciences, Durham, NC) as detailed earlier [16], [17]. Five-micrometer serial sections were cut and stained with hematoxylin and eosin (H&E), and examined for NM-induced histopathological changes that were scored as follows: a) Percent length or area of mouse skin epidermis or dermis, respectively showing the events listed below and b) incidences of scab formation or outgrowths in skin section. The histopathological changes were assessed in the three skin sections [(approx.18 mm length per skin tissue section under 400× magnification using Axiovision Rel 4.5 software (Carl Zeiss, Inc. Germany)] from each mouse and an average value was arrived at for each mouse in each exposure group. The infiltration of inflammatory cells was scored as: +, few (less than 100); ++, moderate (100–1000); +++, high (more than 1000) inflammatory cells per skin section (at 400× magnification). The extravasated RBCs were scored as: +, few (less than 10%); ++, moderate (10–30%); +++, high (more than 30%) extravasated RBCs per skin section (at 400× magnification).

### Statistical Analysis

Data were analyzed using one-way analysis of variance (one-way ANOVA) to get the statistically significant difference in control versus treated groups, with Tukey or Bonferroni t-test for multiple comparisons (SigmaStat 2.03). Differences were considered significant if the *p* value was ≤0.05. Data are presented as the mean ± standard error of mean (SEM).

## Results

Our published study showed that NM exposure (3.2 mg) for 12–120 h caused edema, erythema, microblister formation, pigmentation changes, dry skin and wounded areas on the skin of SKH-1 hairless and C57BL/6 mice [15]. The skin from these mice was collected, fixed, sectioned and H&E stained to further assess for structural changes associated with these NM-induced lesions.

### NM Exposure causes Structural Changes in the Epidermis of the Skin in SKH-1 and C57BL/6 Mice

In the epidermis, death of keratinocytes and epidermal dermal separation, as well as inflammation-related changes with SM exposure were observed [4], [18]. Hence, we sought to study these structural changes following NM exposure in hairless and haired mice.

#### i) Epidermal thickness and microvesication

**Figure 1:**
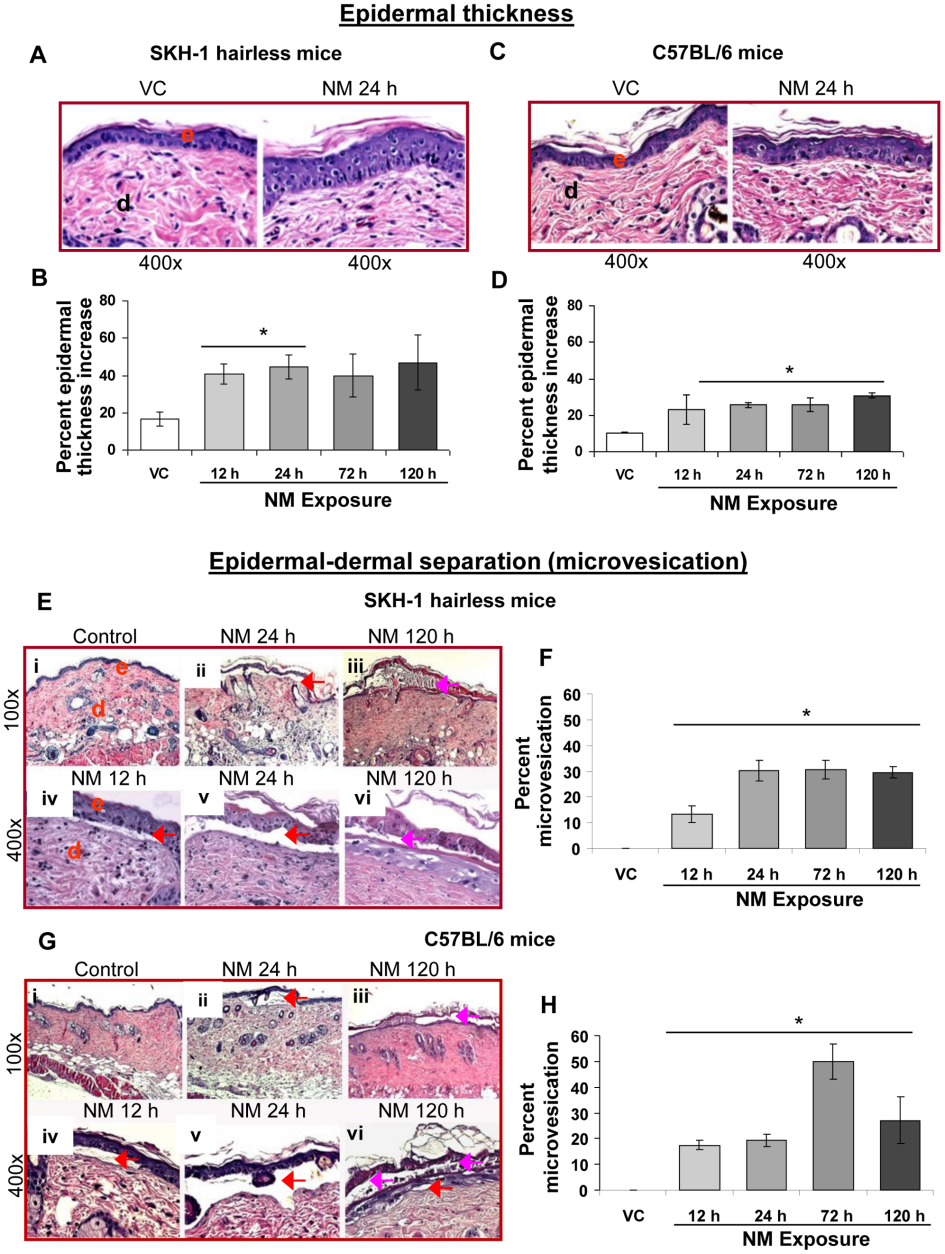
NM exposure causes epidermal thickness and microvesication in the skin of SKH-1 hairless and C57BL/6 mice. Dorsal skin of mice was exposed topically to either 200 µL of acetone or NM (3.2 mg) in 200 µL acetone. After 12, 24, 72 and 120 h of NM exposure, mice were sacrificed and dorsal skin tissue sections (5 µm) were processed, H&E stained and analyzed as detailed under Materials and Methods. Panels A and C are representative H&E stained skin sections (400× magnification) showing epidermal thickness from vehicle control and 24 h exposed NM exposed skin tissue in SKH-1 and C57BL/6 mice, respectively. Panel E (i–iii) and G (i–iii) are representative H&E stained skin sections (100× magnification), and E (iv–vi) and G (iv–vi) are representative H&E stained skin sections (400× magnification) showing microvesication from vehicle control and 12–120 h NM exposed skin tissue in mice. These NM-related histopathological changes were assessed as detailed under materials and methods, and calculated as percent length of mice skin epidermis showing epidermal thickness (B and D) and microvesication (F and H). Data presented are mean ± SEM of 3–5 animals in each group. *, p<0.05 compared to respective vehicle control; VC, vehicle control; NM, nitrogen mustard; e, epidermis; d, dermis; red arrows, microvesication (epidermal and dermal separation); pink arrows, microvesication within the epidermal layer.

Our clinical observations in NM (3.2 mg) exposed haired and hairless mice skin revealed increased bi-fold thickness and microblister like appearance [15]. In this study, the histopathology of the skin sections from these NM-exposed mice showed increased epidermal thickness and epidermal-dermal separation (Fig. 1). NM induced more than a two fold increase in areas (percent length of skin section) of epidermal thickness in both SKH-1 and C57BL/6 mice (Fig. 1B and D). In SKH-1 mice, a significant (p<0.05) NM-induced increase in thickness of skin sections was observed only at 12 and 24 h after exposure (Fig. 1B). However, in C57BL/6 mice, this NM-induced lesion showed a significant (p<0.05) increase after 24, 72 and 120 h of its exposure (Fig. 1D). Our published clinical study showed appearance of small and large areas of yellowish microblisters with swollen skin in both the mouse strains at 12 and 24 h post-NM exposure, respectively. These microblisters covered larger skin area after 72 and 120 h of NM exposure but appeared less swollen and compressed in both these mouse strains [15]. Blister formation on the skin is related to epidermal-dermal separation [7], [10]. Histopathology of skin sections from these NM-exposed mice with microblisters revealed smaller epidermal-dermal separations at 12 h post-exposure, which were more prominent after 24 h of NM exposure (Fig. 1E and G; iv-vi). Apart from epidermal-dermal separation (red arrows), NM-induced separation within the epidermis was also seen (pink arrows; Fig. 1E and G). In SKH-1 mice, 13% length of skin sections showed epidermal-dermal separation at 12 h post-NM exposure, this lesion increased to 30% after 24 h and remained equivalent thereafter at 72 and 120 h post-exposure (Fig. 1F). In C57BL/6 mice, 12 and 24 h NM exposure showed epidermal-dermal separation in less than 20% length of assessed skin sections (Fig. 1H). However, after 72 h of NM exposure, this type of lesion was maximal, which covered 50% length of the observed skin sections (Fig. 1H). Microvesication was not seen in vehicle control skin sections (Fig. 1E–H).

#### ii) Dead epidermal layer, epidermal denuding and ulceration, and scab formation

**Figure 2:**
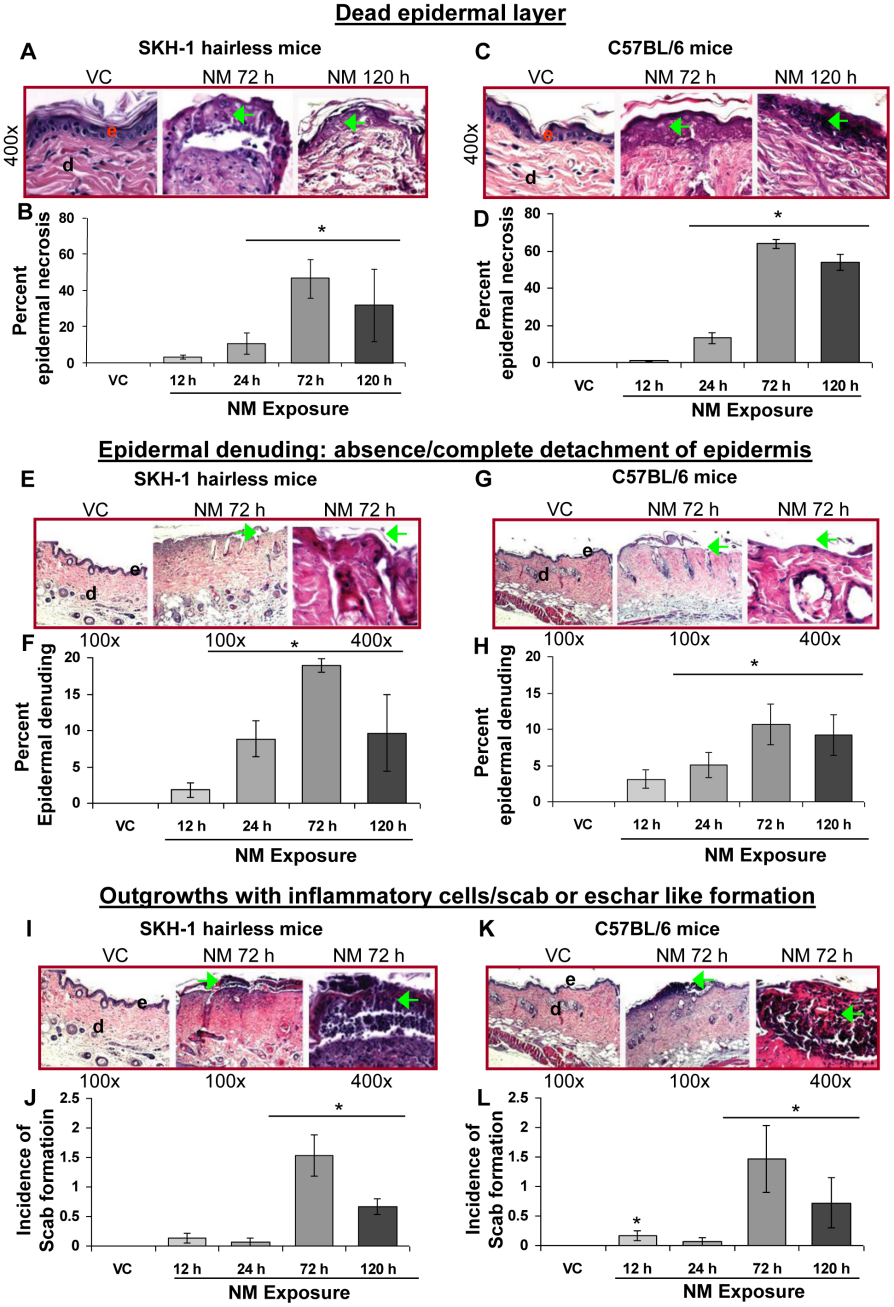
NM exposure causes epidermal cell death, epidermal denuding and ulceration, and scab formation in the skin of SKH-1 and C57BL/6 mice. Dorsal skin of mice was exposed topically to either 200 µL of acetone or NM (3.2 mg) in 200 µL acetone. After 12, 24, 72 and 120 h of NM exposure, mice were sacrificed and dorsal skin tissue sections (5 µM) were processed, H&E stained and analyzed as detailed under Materials and Methods. Panels A and C, E and G and I and K are representative H&E stained skin sections (100 or 400× magnification) showing epidermal cell death, epidermal denuding and scab formation, respectively from vehicle control and 72 or 120 h NM exposed skin tissue in SKH-1 and C57BL/6 mice. These NM-related histopathological changes were assessed as detailed under materials and methods, and calculated as percent length of mice skin epidermis showing epidermal cell death (B and D) epidermal denuding and ulceration (F and H), or as incidences of scab formation/outgrowths (J and L). Data presented are mean ± SEM of 3–5 animals in each group. *, p<0.05 compared to respective vehicle control; VC, vehicle control; NM, nitrogen mustard; e, epidermis; d, dermis; green arrows, necrosis/dead epidermal layer or epidermal denuding or scab formation.

Skin pathology effects after 12–120 h NM (3.2 mg) exposure included erythema, broken skin (ulceration) and dead peeled skin areas [15]. Therefore, we sought to study the structural changes in the skin associated with these NM-related observations. Examination of the NM-exposed skin sections from both hairless and haired mice showed areas of partial and full thickness epidermal death (Fig. 2A and C), epidermal denuding/absence of epidermis and ulcerated skin areas (Fig. 2E and G), and scab or eschar like formation (Fig. 2I and K). NM exposure after 12–72 h in both the mouse strains resulted in a time-dependent increase in the length of examined skin sections with dead, and denuded epidermis. Less of this injury was observed at 120 h after NM exposure (Fig. 2B and D; 2F and H). At 72 h post-NM exposure, dead epidermis was maximal; 46% and 64% length of examined skin sections showed dead epidermis in SKH-1 and C57BL/6 mice, respectively (Fig. 2B and D). Similarly, a maximum of 19% and 5% of the length of these skin sections in SKH-1 and C57BL/6 mice, respectively, showed denudation of epidermis at 72 h after NM exposure (Fig. 2F and H). In addition to dead and denuded epidermal areas, NM exposure also resulted in incidences of small outgrowths/scab formation where the dead epidermal regions accumulated inflammatory cells (Fig. 2I and K; green arrows). Little scab formation was observed prior to 72 h after NM exposure; at this time point it was maximal and then decreased at 120 h post-exposure (Fig. 2J and L). None of these lesions were observed in skin tissue sections from controls (Fig. 2).

#### iii) Parakeratosis and hyperkeratosis

**Figure 3:**
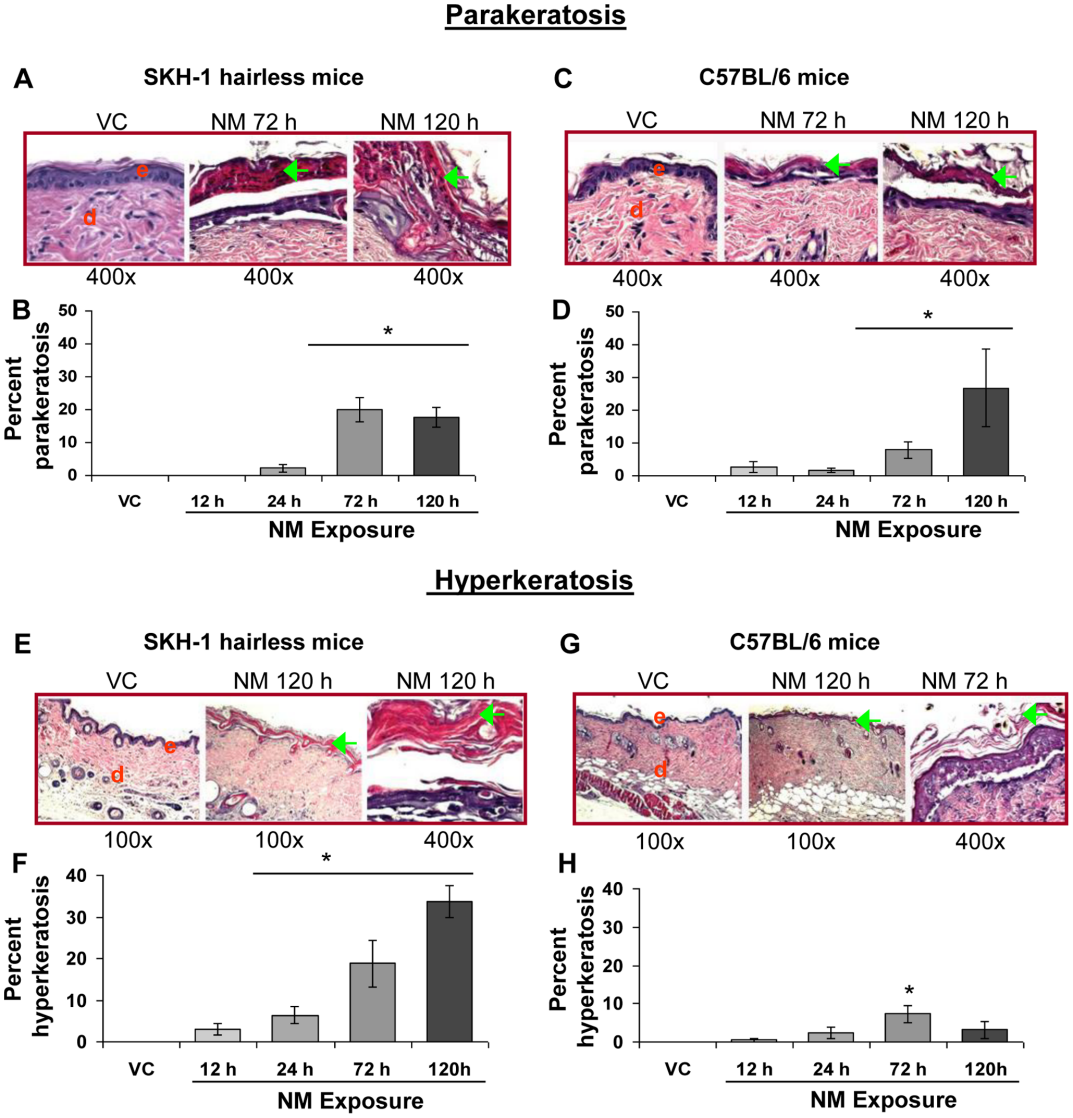
NM exposure causes parakeratosis and hyperkeratosis in the skin epidermis of SKH-1 and C57BL/6 mice. Dorsal skin of mice was exposed topically to either 200 µL of acetone or NM (3.2 mg) in 200 µL acetone. After 12, 24, 72 and 120 h of NM exposure, mice were sacrificed and dorsal skin tissue sections (5 µM) were processed, H&E stained and analyzed as detailed under Materials and Methods. Panels A and C (i–iii) and E and G (i–iii) are representative H&E stained skin sections (100 or 400× magnification) showing parakeratosis and hyperkeratosis, respectively, from vehicle control as well as NM exposed (72 and 120 h) skin tissue in mice. These NM-related histopathological changes were assessed as detailed under materials and methods, and calculated as percent length of mice skin epidermis showing parakeratosis (B and D) and hyperkeratosis (F and H). Data presented are mean ± SEM of 3–5 animals in each group. *, p<0.05 compared to respective vehicle control; VC, vehicle control; NM, nitrogen mustard; e, epidermis; d, dermis; green arrows, parakeratosis, hypercornification or acanthosis.

Gross examination of NM treated hairless and haired mice showed thick leathery, scaly and desquamating skin as well as pigmentation changes at 72 and 120 h post-NM exposure [15]. Parakeratosis refers to abnormal retention of nucleated keratinocytes in the stratum corneum. Hyperkeratosis is overproduction of keratin (could also cause altered pigmentation) and thickening of the stratum corneum [19]. These conditions are reported following SM exposure in mice [10], [11] and could be related to the above mentioned pathological features observed in mice with NM. Accordingly, examination of skin sections from NM exposed skin revealed the occurrence of parakeratosis (Fig. 3A and C, green arrows) and hyperkeratosis (Fig. 3E and G, green arrows). A maximum quantity of parakeratosis was observed at 72 h post-NM exposure in SKH-1 mice (Fig. 3B). However, in C75BL/6 mice it took 120 h of NM exposure to induce a maximal amount of parakeratosis; at this time it occurred in 27% of the length of skin sections (Fig. 3D). A time-dependent increase in hyperkeratosis was evident in SKH-1 mice between 12–120 h post-NM exposure; the maximal length of examined skin sections with hyperkeratosis was 34% observed at 120 h post-exposure (Fig. 3F). In contrast, in C57BL/6 mice, NM-exposure (12–72 h) resulted in a time-dependent increase in the length of skin sections with hyperkeratosis, which only reached a maximum of 7% at 72 h and decreased at 120 h post-NM exposure (Fig. 3H).

#### iv) Acanthosis and hyperplasia

**Figure 4:**
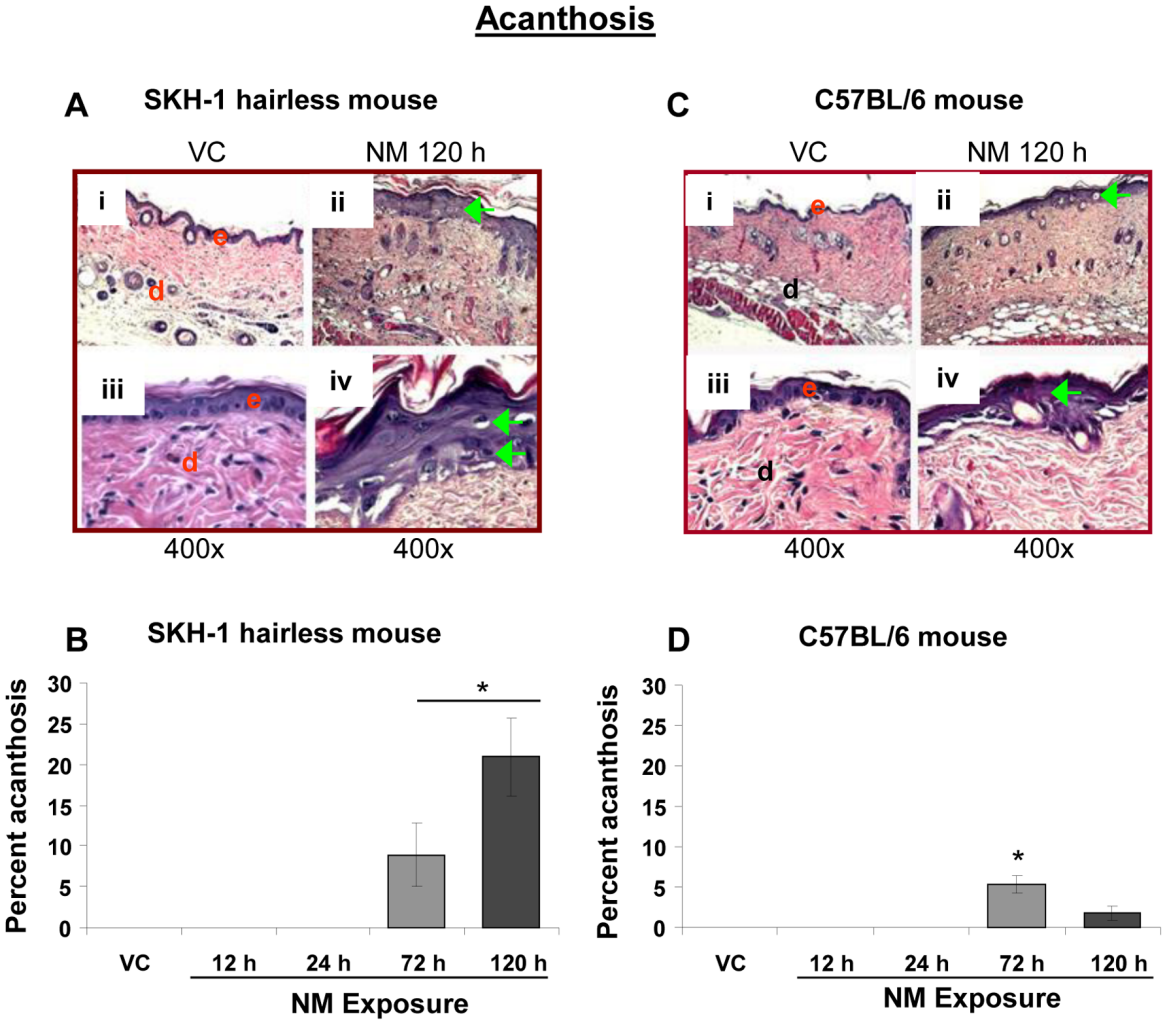
NM exposure causes acanthosis in the skin epidermis of SKH-1 and C57BL/6 mice. Dorsal skin of mice was exposed topically to either 200 µL of acetone or NM (3.2 mg) in 200 µL acetone. After 12, 24, 72 and 120 h of NM exposure, mice were sacrificed and dorsal skin tissue sections (5 µM) were processed, H&E stained and analyzed as detailed under Materials and Methods. Panels A and B are representative H&E stained skin sections (100 or 400× magnification) showing acanthosis from vehicle control as well as NM exposed (72 and 120 h) skin tissue in mice. This NM-related histopathological change was assessed as detailed under materials and methods, and calculated as percent length of mice skin epidermis showing acanthosis (B and D). Data presented are mean ± SEM of 3–5 animals in each group. *, p<0.05 compared to respective vehicle control; VC, vehicle control; NM, nitrogen mustard; e, epidermis; d, dermis; green arrows, acanthosis.

Acanthosis has been reported to be induced by SM; it is defined as diffuse hyperproliferation and thickening of the stratum spinosum with an increased size of the cells in this layer [4], [8]. This lesion was observed at 72 and 120 h post-NM exposure in both SKH-1 and C57BL/6 mice skin sections (Fig. 4). A more profound NM-induced increase in length of skin sections with acanthosis was observed in SKH-1 mice as compared to C57BL/6 mice (Fig. 4B and D; green arrows). This NM-induced lesion was maximal (21% length of skin sections) at 120 h post-exposure in SKH-1 mice (Fig. 4B). However, only a maximum of 5% length of examined C57BL/6 mice skin sections showed acanthosis at 72 h post-NM exposure (Fig. 4D). Acanthosis was not observed in mice from the vehicle control group, nor from 12 and 24 h NM-exposed mice (Fig. 4).

### NM Exposure causes Structural Changes in the Dermis of the Skin in SKH-1 and C57BL/6 Mice

SM exposure causes a profound inflammatory response including dermal edema, necrosis and influx of inflammatory cells in the dermis. Hence, we next assessed these changes following NM exposure in SKH-1 and C57BL/6 mice.

#### i) Dermal necrosis and edema

**Figure 5:**
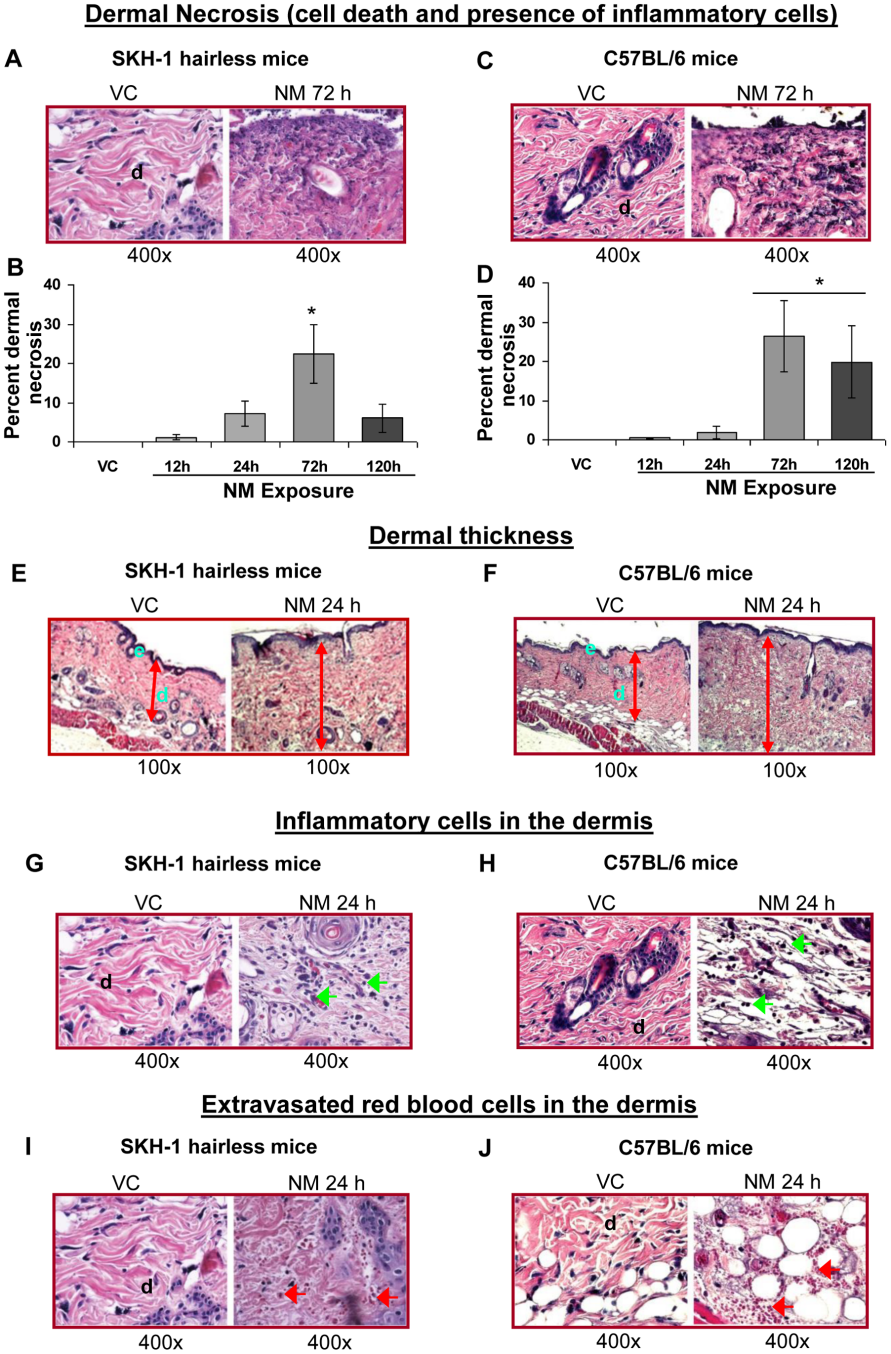
NM exposure causes necrosis, dermal thickness, recruitment of inflammatory cells and extravasated red blood cells in the dermal region of SKH-1 and C57BL/6 mice skin. Dorsal skin of mice was exposed topically to either 200 µL of acetone or NM (3.2 mg) in 200 µL acetone. After 12, 24, 72 and 120 h of NM exposure, mice were sacrificed and dorsal skin tissue sections (5 µM) were processed, H&E stained and analyzed as detailed under Materials and Methods. Panels A and C, E and F, G and H, and I and J are representative H&E stained skin sections from vehicle control as well as NM exposed skin tissue in mice for dermal necrosis (with presence of breaking down inflammatory cells, mainly neutrophils), dermal thickness, inflammatory cells, and extravasated red blood cells. These NM-related histopathological changes were assessed as detailed under materials and methods, and calculated as percent area of mice skin dermis showing necrosis (B and D), inflammatory cells (Table 1), and extravasated red blood cells (Table 2). *, p<0.05 compared to respective vehicle control; VC, vehicle control; NM, nitrogen mustard; d, dermis; green arrows, inflammatory cells; red arrows, extravasated red blood cells.

Histological analysis of NM-exposed skin tissue sections from both hairless and haired mice revealed that NM-induced dermal necrosis (dead dermal areas), which was accompanied by the presence of a large number of inflammatory cells (Fig. 5A and C). In both mouse strains, NM exposure for 12–72 h caused a time-dependent increase in the length of skin sections with dermal necrosis. Inflammation reached a maximum at 72 h and then decreased at 120 h post-exposure (Fig. 5B and D). NM exposure for 72 h resulted in dermal necrosis in 22% and 26% length of skin sections in SKH-1 and C57BL/6 mice, respectively (Fig. 5B and D). NM exposure in both the mouse strains also caused an increase in dermal thickness; this was maximal at 24 h after exposure, and decreased at 72 and 120 h post-exposure (Fig. 5E and F).

#### ii) Infiltration of inflammatory cells and extravasated red blood cells

NM exposure also resulted in an influx of inflammatory cells (Fig. 5G and H) and extravasated red blood cells (RBCs; Fig. 5I and J) in the dermal region in both SKH-1 and C57BL/6 mice. The NM-induced increase in the inflammatory cells was scored as detailed under Materials and Methods. NM exposure-induced a profound infiltration of inflammatory cells at 12, 24 and 72 h post-exposure in SKH-1 mice, which was moderate at 120 h after exposure (Table 1). However, in C57BL/6 mice, a large number of inflammatory cells were observed only at 24 h post-NM exposure (Table 1). Similarly, a large number of extravasated RBCs were observed in the skin sections at 24, 72 and 120 h post-NM exposure in SKH-1 mice and at 12–120 h after its exposure in C57BL/6 mice (Table 2).

**Table 1:** NM exposure caused infiltration of inflammatory cells (PMNs) in the dermis of SKH-1 hairless and C57BL/6 mice following 12–120 h of its exposure.

**SKH-1**					
Exposure	VC	12 h NM	24 h NM	72 h NM	120 h NM
Inflammatory cells	+	+++	+++	+++	++
**C57 BL/6**					
Exposure	VC	12 h NM	24 h NM	72 h NM	120 h NM
Inflammatory cells	+	++	+++	++	++

+, Few (less than 100); ++, moderate (100–1000); +++, high (more than 1000) inflammatory cells per skin section (at 400×); VC, vehicle control.

**Table 2:** NM exposure caused an increase in the percent extravasated RBCs in the dermis of SKH-1 hairless and C57BL/6 mice following 12–120 h of its exposure.

**SKH-1**					
Exposure	VC	12 h NM	24 h NM	72 h NM	120 h NM
Extravasated RBCs	+	++	+++	+++	+++
**C57 BL/6**					
Exposure	VC	12 h NM	24 h NM	72 h NM	120 h NM
Extravasated RBCs	++	+++	+++	+++	+++

+, Few (less than 10%); ++, moderate (10–30%); +++, high (more than 30%) extravasated RBCs per skin section (at 400×); vehicle control.

## Discussion

NM is an analog of SM with reported chemical properties and cutaneous toxic effects similar to SM. Thus far it has not been used as a weapon of mass destruction [14], [20]–[22]. However, NM was stockpiled during World War II and its leakage from Tankers bombed by Germans at Bari Harbor in Italy was reported to cause injuries as well as human casualties [23]. Therefore NM poses a potential warfare and terrorist threat as does SM [21], [24]. Both SM and NM are powerful vesicants and alkylating agents, which are reported to cause edema, blister formation, ulceration, necrosis and desquamation [6], [14], [25]. Consistent with these earlier reports, our published study examining NM exposure in both SKH-1 hairless and C57BL/6 mice identified clinical effects of NM, which are comparable to clinicopathological injuries with SM [15]. In the current study, we further evaluated the histopathological structural changes related to the clinical effects of NM exposure.

Inflammatory response in the skin is associated with an increase in epidermal thickness, which has also been reported following SM exposure [13]. In our study, NM exposure caused comparable increases in epidermal thickness in both hairless and haired mice. This NM-induced lesion could contribute to the NM-caused edema and increased skin bi-fold thickness observed in these mice in our earlier report [15]. Skin blistering is the most prominent characteristic lesion following SM exposure [3], [26]–[28]. Normally the basal epidermal keratinocytes maintain attachment to the basement membrane through hemidesmosomes [10], [28]. However, SM-induces keratinocyte cell death via apoptosis and results in protease digestion of anchoring filaments of the epidermal-dermal junction allowing blister formation [28]. Similar to the blister formation observed following SM exposure, our NM-induced microblisters show a comparable histopathology observed as epidermal-dermal separation associated with epidermal cell death. This is in agreement with reported NM-induced keratin destruction which leads to lysis of basal epidermal cells and blister formation [29]. The NM-induced epidermal-dermal separations in both SKH-1 and C57BL/6 observed in this study are consistent with those observed in SKH-1 mice with NM [30]. In addition, the NM-related epidermal-dermal separations are comparable to the SM-induced epidermal-dermal separations in human and animal (mouse and guinea pig) skin [3], [4], [6], [8], [12], [28], [31], [32].

Following 48–72 h of cutaneous SM exposure in humans, blisters in the skin burst open causing ulceration, epidermal denudation, necrosis and finally scab or eschar like formation [9]. Similarly, histopathological examination of NM-exposed skin sections from hairless and haired mice in the present study revealed dead/necrotic epidermis, epidermal denudation and ulceration, and scab formation which was maximal at 72 h post-NM exposure. Both SM and NM target the basal epidermal keratinocytes [33], and cause apoptotic and necrotic cell death. Consistent with this, NM exposure in this study also induced necrotic/dead epidermis regions in both hairless and haired mice due to basal epidermal cell death. It is suggested that keratinocyte cell death is one of early responses to SM exposure and plays an important role in blister formation, inflammation and the wound healing process [31], [34], [35]. The NM-induced epidermal denuding observed in this study could contribute to the ulceration of the skin. This denudation is also comparable to the epidermal loss/desquamation observed following SM exposure in human skin, and in mouse and guinea pig models [3], [13], [32], [36]. The SM-induced necrotic and ulcerated regions of epidermis are reported to be very sensitive to secondary infections [9]. Since NM also induced denudation of epidermis and ulcerated skin areas in mice, further studies are needed to determine whether secondary infections also occurred in these mice. SM exposure in humans causes eschar or scab formation in the skin 72 h after its exposure; a similar pathology has been reported in animal models [11], [12], [32], [37]. The thickened area in the scab contains dead epidermal cells and an inflammatory infiltrate, mainly neutrophils [11]. Similar to SM exposure in humans, NM exposure for 72 h in hairless and haired mice induced maximal incidences of scab formation containing dead cells and inflammatory cells.

The presence of parakeratosis at 72 and 120 h post-NM exposure in our study is consistent with reports of parakeratosis in human and animal models with SM cutaneous exposure [4], [11], [38]. Parakeratosis refers to retention of nucleated keratinocytes in the stratum corneum, and is linked to inflammatory skin diseases like psoriasis [19]. Psoriasis, pruritus and dermatitis are reported chronic signs of SM exposure [9]; these will need to be examined in our NM-exposed mouse models. An acute complication of SM exposure in humans is hyperkeratosis [3], [4], [8], [39]. This thickening of the stratum corneum layer was also observed following NM exposure in the mouse strains studied here, however, it was more pronounced in our SKH-1 hairless mice than C57BL/6 mice. These findings are in accord with SM-induced hyperkeratosis in animal models including SKH-1 hairless mice and guinea pigs [8], [11]. A major keratin in keratinocytes, K10 (associated with epidermolytic hyperkeratosis), is reported to play an important role in SM-exposed hyperkeratosis observed in SKH-1 hairless mice [11]. The role of this keratin in NM-related hyperkeratosis could be further examined. Our study shows that acanthosis with hyperplasia, larger nuclei and areas of re-epithelialization appear in 72 and 120 h NM exposed hairless and haired mouse skin. These findings are consistent with reports that SM exposure in humans and animal models caused delayed acanthosis and hyperplasia to compensate for cell death and thus allow healing of wounds by forming a new epidermal layer [4], [8], [11], [39], [40].

Dermal changes following SM exposure in humans and animals include edema, infiltration of inflammatory cells, extravasated erythrocytes, necrosis and endothelial swelling [4], [8], [12], [13], [39]. Our assessments in this study also show increases in edema, infiltration of inflammatory cells, dermal necrosis and extravasation of RBCs following 12–120 h of NM exposure. In our study, the dermal thickness peaked at 24 h after NM exposure indicating that this is an early inflammatory response. The inflammatory cells including neutrophils, macrophages and mast cells in the dermis following SM exposure release cytokines and other inflammatory mediators to initiate healing process [4], [11], [17], [41], [42]. Neutrophils, macrophages and mast cells could also be involved in NM-related inflammatory response since NM caused profound infiltration of inflammatory cells in both the mouse strains. We then observed a maximal dermal necrosis at 72 h post-NM exposure.

According to the observation from this study, immunohistochemical analyses, and other specific assays to further investigate the NM-induced inflammatory response, epidermal cell death, and microvesication are under investigation. These studies will further help in dissecting and examining mechanisms involved in NM-induced skin injuries, which will also be useful in screening treatment agents against vesicant injuries.

In summary, this study provides a detailed assessment of NM-related structural changes in both hairless and haired mice to establish that its histopathological parameters are comparable to SM exposure in humans and other animal models. We believe this histopathological analysis will be valuable for screening and identification of therapies against skin injuries induced by both SM and NM.
